# Specific Fluorescence *in Situ* Hybridization (FISH) Test to Highlight Colonization of Xylem Vessels by *Xylella fastidiosa* in Naturally Infected Olive Trees (*Olea europaea* L.)

**DOI:** 10.3389/fpls.2018.00431

**Published:** 2018-04-06

**Authors:** Massimiliano Cardinale, Andrea Luvisi, Joana B. Meyer, Erika Sabella, Luigi De Bellis, Albert C. Cruz, Yiannis Ampatzidis, Paolo Cherubini

**Affiliations:** ^1^Institute of Applied Microbiology, Research Center for BioSystems, Land Use, and Nutrition, Justus-Liebig-University Giessen, Giessen, Germany; ^2^Department of Biological and Environmental Sciences and Technologies, University of Salento, Lecce, Italy; ^3^WSL Swiss Federal Research Institute, Birmensdorf, Switzerland; ^4^Department of Computer and Electrical Engineering and Computer Science, California State University, Bakersfield, CA, United States; ^5^Department of Agricultural and Biological Engineering, Southwest Florida Research and Education Center, University of Florida, Gainesville, FL, United States

**Keywords:** olive quick decline syndrome (OQDS), xylem, vessel occlusion, phytopathogen, fluorescence *in situ* hybridization-confocal laser scanning microscopy (FISH-CLSM)

## Abstract

The colonization behavior of the *Xylella fastidiosa* strain CoDiRO, the causal agent of olive quick decline syndrome (OQDS), within the xylem of *Olea europaea* L. is still quite controversial. As previous literature suggests, even if xylem vessel occlusions in naturally infected olive plants were observed, cell aggregation in the formation of occlusions had a minimal role. This observation left some open questions about the whole behavior of the CoDiRO strain and its actual role in OQDS pathogenesis. In order to evaluate the extent of bacterial infection in olive trees and the role of bacterial aggregates in vessel occlusions, we tested a specific fluorescence *in situ* hybridization (FISH) probe (KO 210) for *X. fastidiosa* and quantified the level of infection and vessel occlusion in both petioles and branches of naturally infected and non-infected olive trees. All symptomatic petioles showed colonization by *X. fastidiosa*, especially in the larger innermost vessels. In several cases, the vessels appeared completely occluded by a biofilm containing bacterial cells and extracellular matrix and the frequent colonization of adjacent vessels suggested a horizontal movement of the bacteria. Infected symptomatic trees had 21.6 ± 10.7% of petiole vessels colonized by the pathogen, indicating an irregular distribution in olive tree xylem. Thus, our observations point out the primary role of the pathogen in olive vessel occlusions. Furthermore, our findings indicate that the KO 210 FISH probe is suitable for the specific detection of *X. fastidiosa*.

## Introduction

*Xylella fastidiosa* is a bacterial phytopathogen that can cause severe diseases in many woody species around the world, such as in grapevine (Pierce’s disease, caused by subsp. *fastidiosa*), citrus (citrus variegated chlorosis or citrus X disease, caused by subsp. *pauca*) and peach (phony peach disease, caused by subsp. *multiplex*) (Chatterjee et al., 2008a). The pathogen can infect more than 350 plant species, including trees of major importance in forest ecosystems and urban greening plantations, such as oak, elm, sycamore, and maple (EFSA, 2016b). Since 2013, a strain named CoDiRO (*Complesso del Disseccamento Rapido dell’Olivo*) caused an epidemic disease, the olive quick decline syndrome (OQDS) that resulted in devastation of 1000s of hectares of olive trees (*Olea europaea* L.) in Salento (Apulia, Italy), including putatively 1000-years-old trees (Saponari et al., 2013; Strona et al., 2017) and infected more than 20 of the region’s other woody or herbaceous species (EFSA, 2016b). Whether or not this strain belongs to the subspecies *pauca* (Cariddi et al., 2014) is controversial, because in a phylogeny-based study its sequence type (ST53) clustered in one clade close but clearly distinct to the subspecies *pauca* (Elbeaino et al., 2015). To date, the most widespread olive cultivars in the region, Cellina di Nardò and Ogliarola di Lecce, showed high susceptibility to the pathogen, while putative resistance was observed for the cultivars Leccino and Frantoio (Giampetruzzi et al., 2016; Martelli, 2016; Luvisi et al., 2017a).

*Xylella fastidiosa* is a xylem-limited species and is transmitted by sap-feeding insect vectors, for example in olive trees mainly by the meadow spittlebug (*Philaenus spumarius* L.) (Saponari et al., 2013). The pathogen invades the xylem, resulting in the blockage of the xylem vessels and consequent restriction of water movement, triggering the related parts of tree crown to dry out (McElrone et al., 2008; Sun et al., 2013). The colonization behavior of the CoDiRO strain within the xylem of the olive tree, economically the most valuable host, is still quite controversial. Xylem vessel occlusions caused by CoDiRO strain in naturally infected *O. europaea* plants have been observed indeed (Cariddi et al., 2014) but never properly characterized and described. In particular, De Benedictis et al. (2017), using PCR-based assay and scanning electron microscopy (SEM), observed that the role played by bacterial cell aggregation in the formation of occlusions is minimal. In contrast, tyloses, gums, and pectin gels were found to be the main cause. Bacterial cells were rarely observed and seemed to never reach concentrations high enough to occlude the vessels. Whereas a similar behavior was found in other species, such as grapevine (Gambetta et al., 2007; Sun et al., 2013; Pèrez-Donoso et al., 2015), Newman et al. (2003) showed that most *X. fastidiosa* green fluorescent cells in symptomatic leaves were present as large colonies and that they caused more severe damage to the plant than small aggregates.

In this study, we aimed at assessing if bacterial cells are accumulated in tracheary elements or not. In order to evaluate the extent of bacterial infection in olive trees and the role of bacterial aggregates in vessel occlusions, we tested a specific fluorescence *in situ* hybridization (FISH) probe for *X. fastidiosa*. The level of infection and vessel occlusion were quantified in both petioles and branches of naturally infected and non-infected olive trees by using confocal laser scanning microscopy (CLSM) and 3D-image modeling.

## Materials and Methods

### Plant Materials and Symptom Assessment

Trials were carried out in two olive tree orchards near the city of Lecce in the Salento region (Apulia, Italy). Olive trees in both orchards were of the same cultivar (“Ogliarola di Lecce”), of about the same age (25–30 years old) and grew under similar soil and climatic conditions and identical management practices. Phytosanitary treatments were carried out since 2014 according to European Union Decision 2015/789, which required the control of the insect vector (*P. spumarius*) through the removal of host plants or applications of chemical treatments. Chemical control of common insects, such as the olive fly (*Bactrocera oleae* Gmelin), was also performed to minimize the possible effects of other pests.

Orchards were considered to be infected by *X. fastidiosa* if all plants have shown OQDS since 2014, which is 1 year after pathogen detection in Apulia, and not infected if the pathogen had not yet been detected in May 2017, when a visual inspection to identify symptomatic trees and to assess the disease severity was done. Pathogen symptoms were recorded and ranked using the following severity scale: 0 = symptomless; 1 = leaf scorching on few branches or few desiccated branches affecting the canopy; 2 = leaf scorching on several branches or desiccation affecting a large part of the canopy; and 3 = canopy with desiccated branches uniformly distributed (Sabella et al., 2018). Because of the widespread occurrence of other pathogens, such as the fungi *Mycocentrospora cladosporioides* (Sacc.) P. Costa ex Deighton and *Spilocaea oleaginea* (Castagne) S. Hughes, and of the bacterium *Pseudomonas savastanoi* pv. *savastanoi* (Smith and Petri), we had to collect samples from trees that showed these diseases in the canopy. However, we collected only branches and leaves that did not show symptoms induced by these pathogens. One week after visual inspection of the trees’ canopies, plants were sampled for qPCR analysis.

### DNA Extraction and qPCR Analysis

The plant material for relative qPCR assay was collected form five symptomatic and three asymptomatic trees. Quantification of the presence of the pathogen was carried out in both the leaves and the branches. Approximately 1 g of leaf petioles (a pool sample from 30 leaves collected from six branches) or vascular bundles (a pool sample from six branches) was transferred to an extraction bag (BIOREBA, Switzerland) and 4 ml of extraction buffer (0.2 M Tris – HCl pH 9, 0.4 M LiCl and 25 mM EDTA) were added. Samples were homogenized using a semi-automatic homogenizer (Homex 6, BIOREBA) at 50% maximum speed. DNA extraction was performed following the protocol of Edwards et al. (1991). Briefly, the DNA solution is first extracted with a phenol-chloroform–isoamyl alcohol mixture to remove protein contaminants and then precipitated with 100% ethanol.

The TaqMan quantitative PCR protocol with XF-F/R primers and XF-P probe (Harper et al., 2010) was used for confirming the contamination of the samples with *X. fastidiosa*. The qPCR reactions were performed in a real-time thermal cycler (ABI PRISM 7900HT, Applied Biosystems, Foster City, CA, United States). Each reaction consisted of 5 μL of 20 ng μL^-1^ DNA extracted from 1 g of leaf petioles, 12.5 μL of Master Mix (Applied Biosystems), 400 nM each forward and reverse primers, 200 nM TaqMan probe ultrapure DNase/RNase-free water (Carlo Erba Reagents, Italy) in a total final volume of 25 μL. Cycling conditions were: initial denaturation (95°C for 10 min), followed by 40 cycles of 95°C for 15 s and 60°C for 1 min, with the final dissociation at 95°C for 15 s, 60°C for 30 s, and 95°C for 15 s. Data analyses and Ct calculations were carried out using the software SDS 1.2 (Applied Biosystems). The relative amount of bacteria was determined by amplification of partial *rimM* open reading frame of *X. fastidiosa* (Harper et al., 2010) relative to the leaf or branch tissue (Sabella et al., 2018). The 2^-ΔCt^ method of relative quantification (semi-quantitative) was used to determine the fold change (FC) of the *X. fastidiosa* populations (Livak and Schmittgen, 2001).

### FISH Probe for Detecting *X. fastidiosa*

A *X. fastidiosa*-specific sequence targeting the 16S rRNA at position 78 (*E. coli* numbering), previously used for microarray (5′-GCCACCCATGGTATTACTACC-3′) (Franke-Whittle et al., 2005), was tested for suitability as specific FISH probe. To prove specificity, *Xanthomonas translucens* M_cs_CA1 (16S rRNA gene sequence Accession No. MF664204; Rahman et al., 2018) was chosen as suitable non-target species, since it showed one single mismatch in the target sequence of the probe (5′-GCCACCCATGGTATT**G**CTACC-3′) according to ProbeCheck search (Loy et al., 2008). FISH was performed on paraformaldehyde-fixed cells at 10, 20, and 30% formamide onto poly-L-lysine coated glass slides, using a 50 ml screw-cap tube as humid chamber. Same than for the *X. fastidiosa*-contaminated plant material, both the Cy5-labeled KO 210 and the mixture of Cy3-labeled universal bacterial probes EUB338, EUB338II, and EUB338III (hereafter EUB338MIX) (Amann et al., 1990; Daims et al., 1999) probes were applied simultaneously. Using the software Las X (Leica Microsystems, Mannheim, Germany), the signal intensity of the two probes was recorded from individual cells (*N* = 40, from five representative images) per each formamide concentration, after subtraction of the average intensity obtained with the Cy3- and Cy5-labeled NONEUB probes (*N* = 15 from three representative images).

### FISH-CLSM Analysis

To be sure that vessel occlusions were quantified by CLSM in symptomatic leaves and in the corresponding branches of qPCR-positive trees, leaves were analyzed by the X-FIDO Deep Learning vision-based program (Cruz et al., 2017). This program is trained to detect symptoms of OQDS and discovers low-level features from raw data to automatically detect veins and colors that lead to symptomatic leaves via machine learning. Confidence scores for each of the three main categories, i.e., *X. fastidiosa*, other diseases and healthy plant, were calculated by the software. The score ranges from 0 to 1, with the higher values indicating the confidence of classification within the category. The same approach was carried out to select asymptomatic leaves and the corresponding branches in qPCR-negative trees. Thus, FISH analysis was carried out on leaves selected by X-FIDO.

Five infected and five non-infected olive trees were analyzed by FISH-CLSM. Several petioles and branches (∼1.5 cm × 1.5 cm) were excised with sterile razor blades, surface sterilized for 1 min in 70% ethanol and then rinsed three times in sterile distilled water. The cuttings were fixed in 4% paraformaldehyde in phosphate-buffered saline (1x PBS, hereafter PBS) overnight at room temperature, followed by washing in PBS buffer for 10 min at room temperature. After fixation, samples were dehydrated by two successive 1-h incubations in each of 70, 80, 95, and 100% ethanol, then embedded in paraffin and cut into 10–50 μm-thick sections with a microtome Leica RM 2155 (Leica Microsystems, Mannheim, Germany). Sections were transferred to 1:1 (v/v) PBS:96% ethanol and maintained at -20°C until FISH staining.

To dissolve the paraffin, sections were embedded in toluene for 3 min at 43°C. After removing the toluene, in-tube FISH staining was performed according to Cardinale et al. (2008): the samples were rinsed with PBS and permeabilized by incubation for 10 min at room temperature in 0.5 mg mL^-1^ lysozyme. After dehydration by ethanolic series (50–70–96%, 3 min each), hybridization was performed at 42°C for 120 min with the Cy3-labeled EUB338MIX and the Cy5-labeled *X. fastidiosa*-specific KO 210 probe, followed by 10 min washing at 43°C. After final rinsing with ice-cold water, the Citifluor AF1 antifade reagent (Linaris Biologische Produkte GmbH, Dossenheim, Germany) was directly dropped into the Eppendorf tube; then the last 1.4–1.7 cm of a 200 μL-micropipette tip were cut and with this cut tip the antifade reagent together with the stained sections were pipetted onto a glass slide and carefully adjusted with forceps under the binocular, in order to avoid both overlap and folding of the sections. Finally, a coverslip was carefully placed on the sections. This modification was necessary because the transfer of the sections from the Eppendorf tube to the glass slide using forceps and the sample drying with compressed air caused cracks and breakage of the sections. For negative controls, a mixture of Cy3- and Cy5-labeled NONEUB FISH probe (Wallner et al., 1993) was used on contaminated material.

Samples were stored up to 4 days in the dark at 4°C until confocal microscopy observations using either a Leica SP5 or a Leica SP8 confocal system (Leica Microsystems, Mannheim, Germany). Cy3 and Cy5 were excited with the laser lines 561 and 633 nm, respectively. Plant tissue was excited additionally with the laser line 405 nm, to induce autofluorescence. Emission was detected in the range 570–613 nm for Cy3, 640–680 nm for Cy5 and 412–469 nm for the plant autofluorescence. The following color codes were assigned to the respective FISH probe signals: Cy3- red, Cy5- green, plant autofluorescence- cyan. An appropriate number (50–80) of optical slices were acquired with a Z-step of 0.5–0.8 μm, and the resulting “confocal stacks” were analyzed with Imaris 8.3.1 (Bitplane, Zurich, Switzerland). Three-dimensional reconstructions were obtained by replacing the original signal with iso-surfaces. ImageJ (Abramoff et al., 2004^1^) and Adobe Photoshop CS6 (Adobe Systems, Inc., United States) were used to assemble and label the final figures.

## Results

### KO 210 FISH Probe Results

Higher concentrations of the nucleic-acid denaturant formamide generally increase stringency of probe hybridization. However lower concentrations are preferred as they might lead to higher fluorescence values (Santos et al., 2014). The KO 210 FISH probe did not give any signal with the non-target species *Xanthomonas translucens* (which has only one mismatch in the probe target sequence) already at 10% formamide, while the EUB338MIX probe always conferred a strong signal (**Supplementary Figure S1**). This demonstrates that KO 210 probe is suitable for the specific detection of *X. fastidiosa*. Additional non-target species showing the same mismatch are shown in **Supplementary Table S1**.

### FISH-CLSM Analysis of Petioles

**FIGURE 1:**
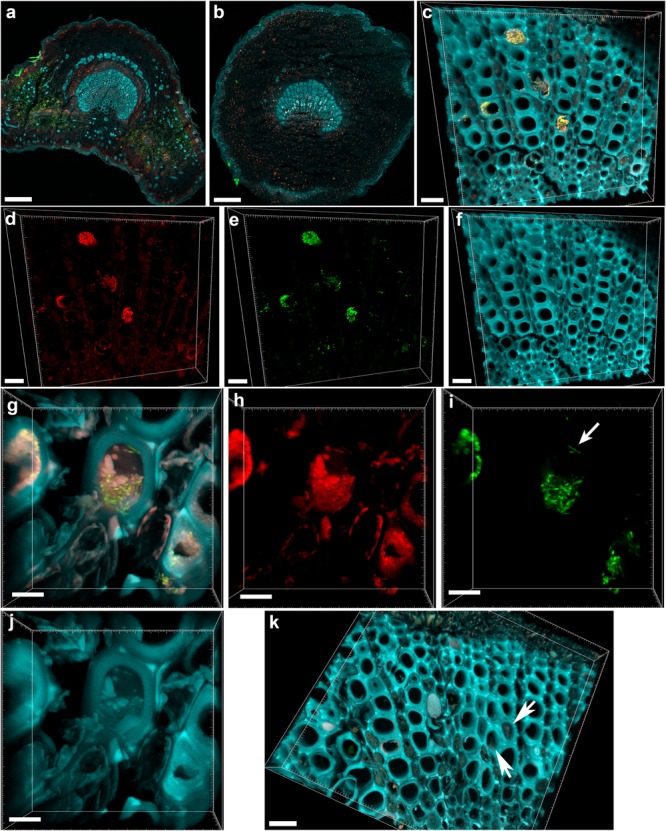
Confocal microscopy images showing the colonization of olive petioles by fluorescence *in situ* hybridization (FISH)-stained *Xylella fastidiosa*. **(a,b)** Maximum projections of confocal stacks showing basal leaf blade and petiole, respectively. **(c)** Magnification of the petiole vessels in the central part of section **(b)**; yellow: *X. fastidiosa*, cyan: plant tissue autofluorescence; **(c)** is the overlap of **(d–f)**. **(d,h)** Signal of the universal bacterial Cy3-labeled EUB338MIX FISH probe (red). **(e,i)** Signal of the *X. fastidiosa*-specific Cy5-labeled KO 210 FISH probe (green); arrow indicates a single *X. fastidiosa* cell with the typical rod-shaped morphology. **(f,j)** Autofluorescence of the plant tissue (cyan). **(g)** Particular of a *X. fastidiosa*-infected vessel showing bacteria (yellow) and extracellular matrix (pink); **(g)** is the overlap of **(h–j)**. **(k)** FISH negative control of an infected petiole section, stained with Cy3- and Cy5-labeled nonsense probes NONEUB; arrows indicate the autofluorescence of occluded vessels (scale bars: 200 μm in **a,b**, 20 μm in **c–f,k**, 5 μm in **g–j**). These are representative images from several observations on five different infected trees.

**FIGURE 2:**
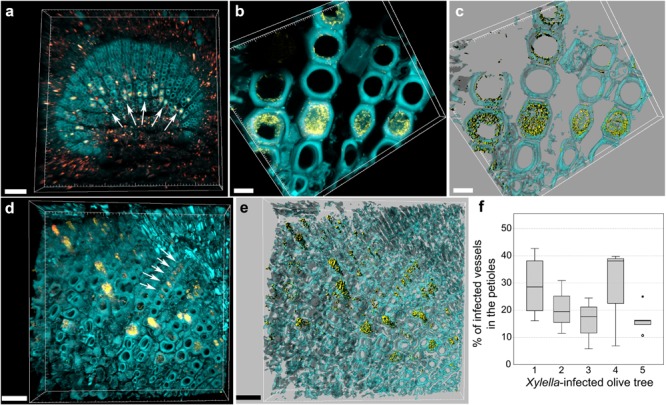
Confocal microscopy images showing the colonization of olive petioles by FISH-stained *Xylella fastidiosa*. **(a,b)** Volume renderings showing the colonization of adjacent, larger vessels by *X*. *fastidiosa*, which often resulted in a characteristic “infection hemi-ring” (arrows). **(c)** Three-dimensional model of **(b)**. **(d)** Heavily infected samples showed also a radial colonization pattern (arrows). **(e)** Three-dimensional model of **(d)**. Yellow: FISH-stained *X*. *fastidiosa* (same color combination of **Figure 1**); cyan: plant tissue autofluorescence. **(f)** Percentage of occluded vessels in petioles and basal leaf blades of *X*. *fastidiosa*-infected olive trees (scale bars: 70 μm in **a**, 10 μm in **b,c**, 30 μm in **d–f**). These are representative images from several observations on five different infected trees.

**FIGURE 3:**
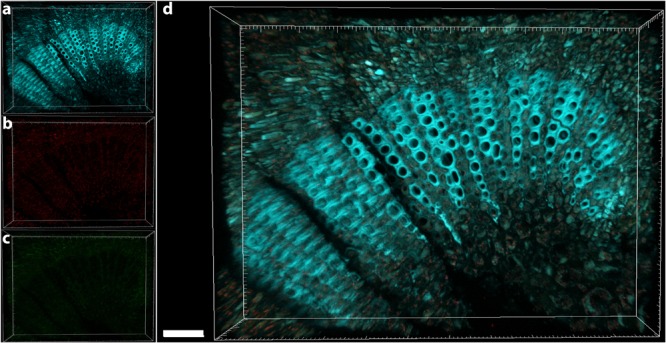
Confocal microscopy images showing the FISH-stained petiole section of an asymptomatic tree. **(a)** Volume rendering showing the plant tissue autofluorescence. **(b)** Signal of the Cy3 (no FISH-conferred signal detected). **(c)** Signal of the Cy5 (no FISH-conferred signal detected). **(d)** Overlap of **(a–c)** (scale bar: 40 μm). These are representative images from several observations on five different uninfected trees.

The central part of the petiole sections showed well-differentiated transport vessels (**Figures 1a,b**). All symptomatic petioles showed colonization and occlusion by *X. fastidiosa* (**Figure 1c**), especially in the larger (and older) innermost vessels. The EUB338MIX FISH probe clearly gave a staining signal, although some plant autofluorescence also appeared in the same emission range (**Figure 1d**). The Cy5-labeled *X. fastidiosa*-specific KO 210 probe showed a very bright signal with an excellent signal-to-noise ratio (**Figure 1e**); the plant tissue autofluorescence was suitable to visualize the plant anatomy (**Figure 1f**). In several cases, the vessel appeared completely occluded by a biofilm containing bacterial cells and extracellular matrix (**Figures 1g,j**). The cells showed the typical *X. fastidiosa* rod-shaped morphology (**Figure 1i**, arrow). FISH negative control of infected sections showed no fluorochrome signal (**Figure 1k** and **Supplementary Figure S2**), but it was possible to clearly identify the occluded vessels by the autofluorescence of the extracellular matrix (**Figure 1k**, arrows; compare to **Figure 1f**). The frequent colonization of adjacent vessels suggested a horizontal movement of the bacteria through the pits connecting the whole vascular system, which often ended up with a typical “infection hemi-ring” [**Figures 1b, 2a** (arrows), **b,c**]. However, radial colonization pattern was also observed in the more severely contaminated samples [**Figures 2d** (arrows), **e**]. In average, the infected plants had 21.6 ± 10.7% of petiole vessels colonized by *X. fastidiosa*, based on 20 representative confocal stacks (2,821 visually checked vessels in total), from five different trees and four independent FISH staining; no significant differences were found between individual trees (ANOVA, *p* = 0.31; **Figure 2f**). No infected or occluded vessels were observed in petiole sections of asymptomatic plants (**Figure 3**).

**FIGURE 4:**
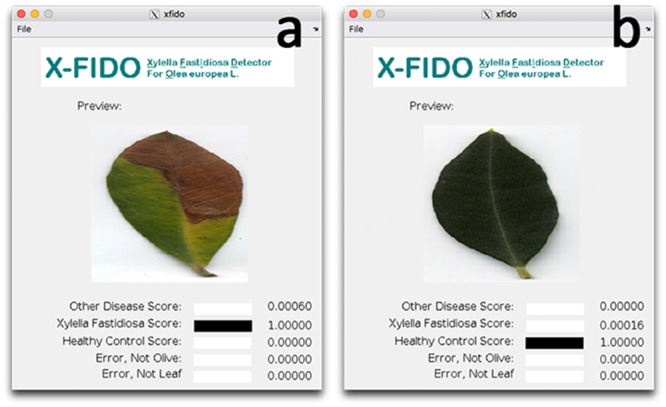
Screen shots of the X-FIDO analysis. **(a)** Detection of symptoms of olive quick decline syndrome (OQDS) due to *Xylella fastidiosa* infection in olive tree. **(b)** Asymptomatic (healthy) leaf.

In order to process with FISH on symptomatic leaves and corresponding branches, mean disease severity, mean qPCR cycle threshold (C_t_) and relative levels of *X. fastidiosa* expressed as fold changes (calculated using leaf tissue as baseline) and image analysis using a deep learning vision-based program (X-FIDO) were assessed. Disease symptoms appeared severe before sampling, scoring a mean value of 2.1 ± 0.3 out of 3 for trees located in *X. fastidiosa*-infected orchards, while almost no symptom of wilting (0.1 ± 0.1) was observed in trees located in *X. fastidiosa*-negative orchards. Leaf symptoms were also confirmed (**Figures 4a,b**) by the X-FIDO software, with scores clearly indicating symptomatic classification of leaves. All tested leaves collected from symptomatic canopies scored the maximum value (1) for classification as *X. fastidiosa*-related symptoms, strongly indicating that all symptomatic canopies had *X. fastidiosa*. In contrast, healthy leaves had a null score, and leaves that were diseased or stressed by other factors than *X. fastidiosa* also had a null score. Conversely, leaves collected from asymptomatic canopies scored maximum values for classification as healthy leaves, with null values for other classifications. Furthermore, all symptomatic samples collected in *X. fastidiosa*-infected areas tested positive with the qPCR. The C_t_ values from the leaf assay samples was 23.6 ± 0.5, while in branches the C_t_ was set at 28.7 ± 0.5. FC in branches was 38.4 ± 14.3 time higher than leaves, suggesting statistically significant higher concentration of the pathogen in the leaf petioles than wood. In contrast, the leaves and branches of asymptomatic samples collected from orchards where the pathogen had not yet been detected tested negatively.

### FISH-CLSM Analysis of Branches

**FIGURE 5:**
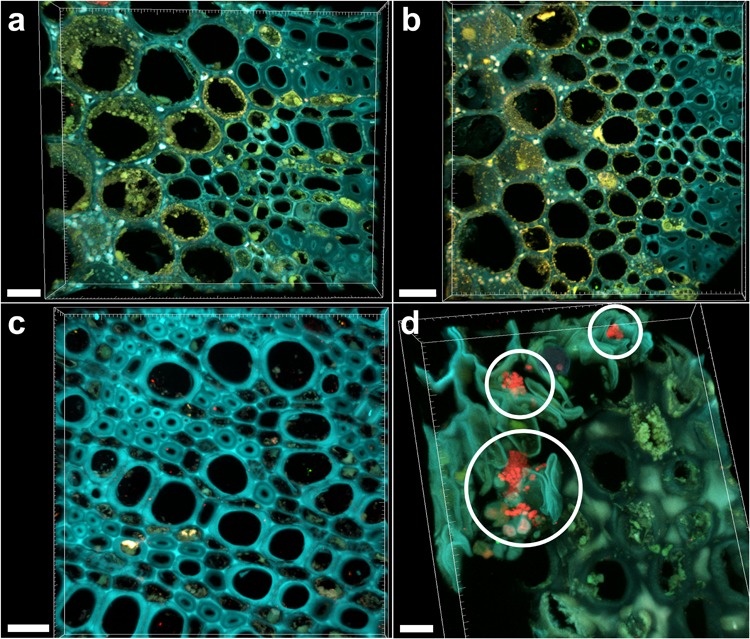
Confocal microscopy images showing FISH-stained branch sections. **(a)** Volume rendering showing the branch section of a *Xylella fastidiosa*-infected olive tree stained by FISH with the universal bacterial Cy3-labeled EUB338MIX and the *X. fastidiosa*-specific Cy5-labeled KO 210 FISH probes: all signals are autofluorescence. **(b)** Negative control of the branch section of a *X. fastidiosa*-infected olive tree stained by FISH with the Cy3- and Cy5-labeled nonsense probes NONEUB. **(c)** Branch section of an asymptomatic olive tree stained by FISH with the Cy3-labeled EUB338MIX and the Cy5-labeled KO 210 FISH probes: no occlusion of xylem vessels was observed. **(d)** Micro-colonies of other bacteria (not *X. fastidiosa*) detected in the branch sections of an infected olive tree, stained by the Cy3-labeled EUB338MIX probe (red; circles) (scale bars: 20 μm in **a,c**, 30 μm in **b**, 10 μm in **d**). These are representative images from several observations on five different infected trees.

Sections of 1-year-old infected branches showed clear occlusions (**Figure 5a**), which did not include bacteria. In fact, FISH negative controls (stained with nonsense probes) showed the same signals (**Figure 5b**). This data could be linked to the high C_t_ (>28) and differences in FC calculated in branches compared to leaves, suggesting a concentration of bacteria in wood samples low to such an extent that it is hard their detection by microscopy. Such occlusions were not observed in asymptomatic trees (**Figure 5c**). The fraction of occluded vessels in branches of symptomatic trees was 50.2 ± 4.3%, thus higher than in the petioles. To be noted that the extracellular material causing the occlusions clearly invaded the lateral connections between adjacent xylem vessels (**Supplementary Figure S3**, arrows). Micro-colonies of other bacteria were also rarely detected in branch sections of infected trees (**Figure 5d**, circles).

## Discussion

A *Xylella*-specific FISH probe was optimized and used in this work to survey the colonization of olive trees under natural conditions. *In situ* visualization is a method complementary to PCR-based assays, which leads to more robust and reliable results and conclusions. Thus, this specific probe represents a new valuable tool for studies of *Xylella*, either under field or greenhouse or laboratory conditions. In grapevine, only a small proportion of the vessels colonized by *Xylella* are completely blocked. In the majority of the *Xylella*-colonized vessels, only a few bacterial cells or small aggregates are seen, suggesting that vessel blockage is not a colonization strategy of *Xylella*, but rather a consequence of endophytic colonization (Newman et al., 2003). The same colonization behavior has been observed for CoDiRO strain in olive trees (De Benedictis et al., 2017). Our findings in branches are similar to what was reported for olive trees, i.e., rare and solitary bacterial cells in the xylem vessels (De Benedictis et al., 2017). Differently from De Benedictis et al. (2017), however, we found vessels blocked by large *X. fastidiosa* aggregates clearly visible in xylem petioles of symptomatic plants. The reason of this higher cell concentration of the pathogen in the leaf petioles (also coherent with the higher C_t_ values from the qPCR assay of branch samples compared to the leaves) could be explained by the higher number of xylem vessels in the petioles (Hopkins, 1981). It is also possible that the insect vectors preferentially feed on petioles than on branches, since the epidermis is much thinner compared to the cork, thus making easier to reach the xylem-sap. Consequently, the pathogenic bacteria could be released directly in the xylem-rich petioles. However, to the best of our knowledge, no experimental or observational evidence is available to support this hypothesis in olive tree. More research is needed to understand the reasons for such difference, which might be crucial for the development of an effective disease control.

Similar observations of vessel obstruction were reported for Pierce’s disease (Tyson et al., 1984; Newman et al., 2003; Chatterjee et al., 2008b). For example, Tyson et al. (1984) and Chatterjee et al. (2008b) observed 19.5–22.5 and 19% of colonized petioles vessels in grapevine, respectively. These values are similar to those observed by FISH, while the high variability of vessel blockage confirms the irregular distribution of the bacterium in olive tree xylem and provides further evidences in support of previous studies of grapes and citrus, where petioles instead of stems appear to be the preferred plant tissue for growth of *X. fastidiosa* (Hopkins, 1981; Baccari and Lindow, 2010; Niza et al., 2015). However, the confocal series are composed of many optical sections, typically 50–80, representing a section of about 30–50 μm thickness. A thicker section is not suitable, because it would interfer too much with the light path, eventually masking the deepest probe signals. It is reasonable to imagine that, considering a larger section, perhaps the whole petiole, a higher number of vessels will be actually blocked.

In our infected samples, vessels lumina appeared to be partially or completely filled and occluded by tyloses, i.e., organic deposits containing various organic and mineral compounds, such as starch, gums, resins, crystals, and phenolic compounds (for a review, see De Micco et al., 2016). Such tyloses, first observed in 1686 by Malpighi and later better described in 1845 by Hermine von Reichenbach (both cited in Zimmermann, 1979), are outgrowths of neighboring parenchyma cells, arising mostly from ray parenchyma cells, which expand into the adjacent vessel through pits. They are formed after wounding to limit the spread of pathogens (Gerry, 1914; Pearce, 1996) and recently were found to be the result of the cessation of water conduction, induced by embolism, i.e., when vessels are air-filled, as for example after pathogen infection (Brodersen et al., 2010). The presence of tyloses affects water movement in living trees and is responsible for a decrease in hydraulic conductivity (Davison, 2014) inducing the death of crown branches. This plant reaction, clearly visible by FISH-CLSM in branches (**Figure 5a**), may also contrast the survival of those *X. fastidiosa* colonies that trigger the occlusion process, which are no more detectable by FISH (**Figure 5**). However, the fraction of such occluded vessels in branches was higher than in the petioles, suggesting an important role in the disease progression. Here, we calculated about 50% of occluded vessels, which is in agreement with previous observations in susceptible olive trees (De Benedictis et al., 2017). This value is also coherent with the situation in grapevine, where the reported fraction of occluded vessels was approximately 60% (Sun et al., 2013) and 30–40% (Krivanek et al., 2005).

In this work, we didn’t test different levels of disease severity, since we had two sample groups: asymptomatic and symptomatic trees, the latter showing similar symptoms. It would be interesting to analyze a gradient of disease severity and link this to the colonization extent of xylem vessels by *Xylella* and by organic compounds. Furthermore, analysis with pure cultures should confirm the specificity of this method and its applicability as diagnostic test.

Our observations underline the primary role of the bacteria in causing vessel occlusion in leaves, and the frequent colonization of adjacent vessels confirms the lateral movement behavior of the bacteria also in olive trees. These data may support the verification of Koch’s postulate, which confirmed the role of *X. fastidiosa* as a causal agent of the disease (EFSA, 2016a). In addition, the assessment of pathogen behavior in hosts that we provide in this analysis can support the knowledge of OQDS pathogenesis and resolve the uncertain role of *X. fastidiosa* in disease development, an uncertainty which has slowed the success of pest management since 2013 (Abbott, 2016; Luvisi et al., 2017b). This is of outmost importance, as disease management to minimize the presence of *X. fastidiosa* in Southern Italy is urgently needed.

## Author Contributions

MC, AL, LDB, and PC designed the research. MC, AL, JBM, ES, AC, and YA performed the research. JBM and ES analyzed the data. MC, AL, LDB, JBM, and PC wrote the paper.

## Conflict of Interest Statement

The authors declare that the research was conducted in the absence of any commercial or financial relationships that could be construed as a potential conflict of interest.

## Footnotes

1 https://imagej.nih.gov/ij/

## Supplementary Material

FIGURE S1 Test for specificity of the FISH KO 210 probe toward *Xylella fastidiosa*. **(a)** Cells of *Xanthomonas translucens* M_cs_CA1 (having only one mismatch in the target sequence of the probe) were not hybridized, already at 10% of formamide. Simultaneous hybridization with the universal bacterial probe EUB338MIX (red) was successful with all formamide concentrations. For the calculation of the intensity units shown in, the average values obtained with nonsense FISH probes (NONEUB, **b**) were subtracted from the values of probes EUB338MIX and KO 210 **(c)**.

FIGURE S2 Confocal microscopy images, showing the FISH negative control of the basal leaf blade section of a *Xylella fastidiosa-*infected olive tree. **(a)** Volume rendering showing only autofluorescence (including the autofluorescence of occluded vessels). **(b)** Magnification of **(a)**, overlap of **(c–e)**. **(c)** Signal of the Cy3–NONEUB FISH probe. **(d)** Signal of the Cy5-NONEUB FISH probe. **(e)** Plant tissue autofluorescence (scale bars: 150 μm in **a**, 50 μm in **b**–**e**). These are representative images from several observations on five different infected trees.

FIGURE S3 Volume rendering of a confocal microscopy stack, showing the autofluorescence of the branch section of a *Xylella fastidiosa*-infected olive tree. **(a)** Xylem vessels (cyan) are occluded by an extracellular matrix (red); **(a)** is the overlap of **(b,c)**. **(b)** Autofluorescence of the plant vessels. **(c)** Autofluorescence of the extracellular matrix, which also invaded the lateral connections between adjacent vessels (arrows). **(d)** Three-dimensional reconstruction of **(c)**. (Scale bars: 20 μm). These are representative images from several observations on five different infected trees.

TABLE S1 Alignment between the sequence of the FISH probe KO 210 and all target organisms, as well as all one-mismatch-only, non-target organisms (according to BLAST and ProbeCheck search).
