# Supplementary figures and images for: Specific Fluorescence in Situ Hybridization (FISH) Test to Highlight Colonization of Xylem Vessels by Xylella fastidiosa in Naturally Infected Olive Trees (Olea europaea L.)
Source: Front Plant Sci. 2018 Apr 6;9:431. doi: 10.3389/fpls.2018.00431 (PMC5897508; doi:10.3389/fpls.2018.00431)

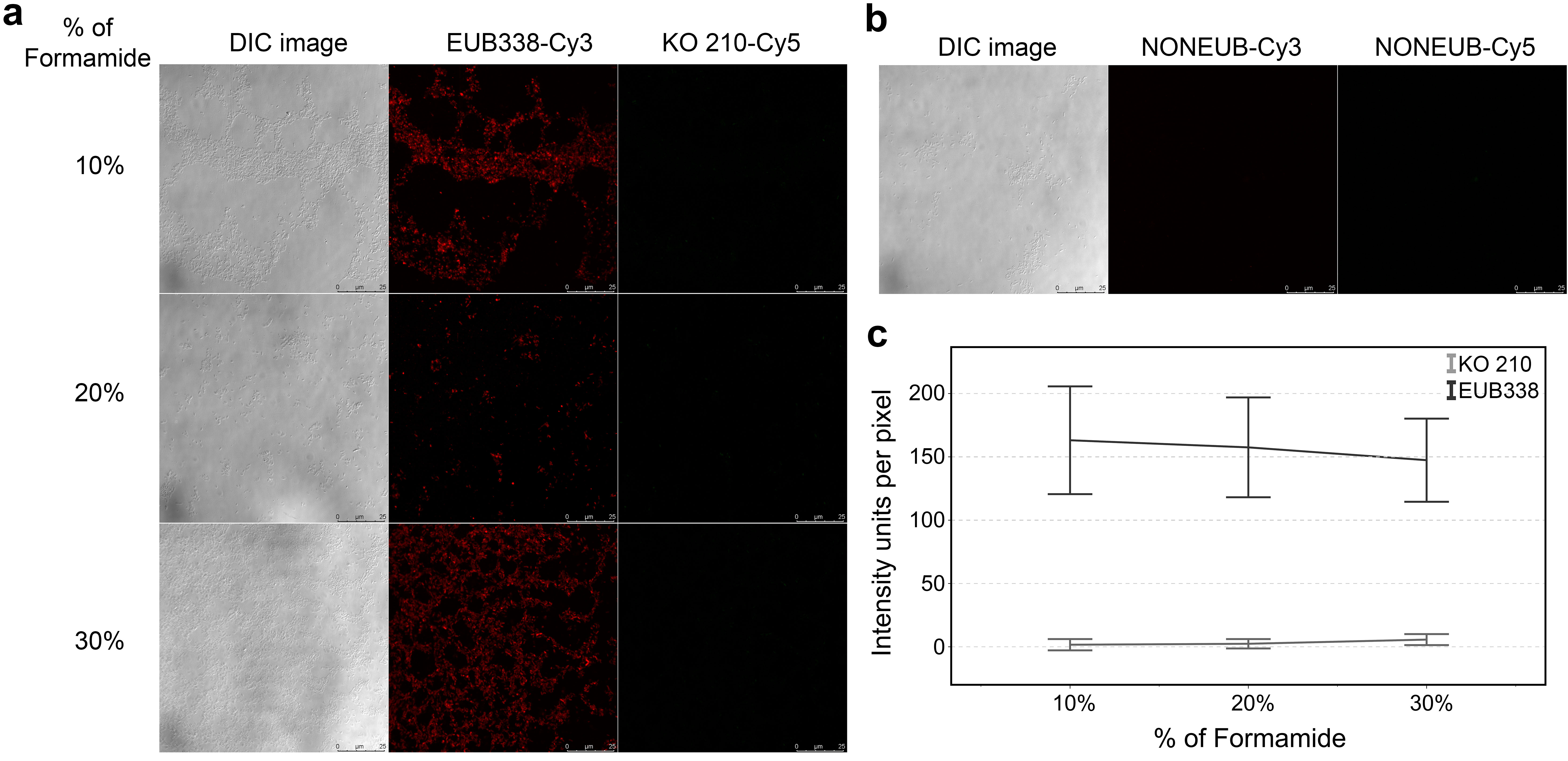

Supplement: FIGURE S1 — Test for specificity of the FISH KO 210 probe toward Xylella fastidiosa. (a) Cells of Xanthomonas translucens M_cs_CA1 (having only one mismatch in the target sequence of the probe) were not hybridized, already at 10% of formamide. Simultaneous hybridization with the universal bacterial probe EUB338MIX (red) was successful with all formamide concentrations. For the calculation of the intensity units shown in, the average values obtained with nonsense FISH probes (NONEUB, b) were subtracted from the values of probes EUB338MIX and KO 210 (c). [file Image_1.JPEG]

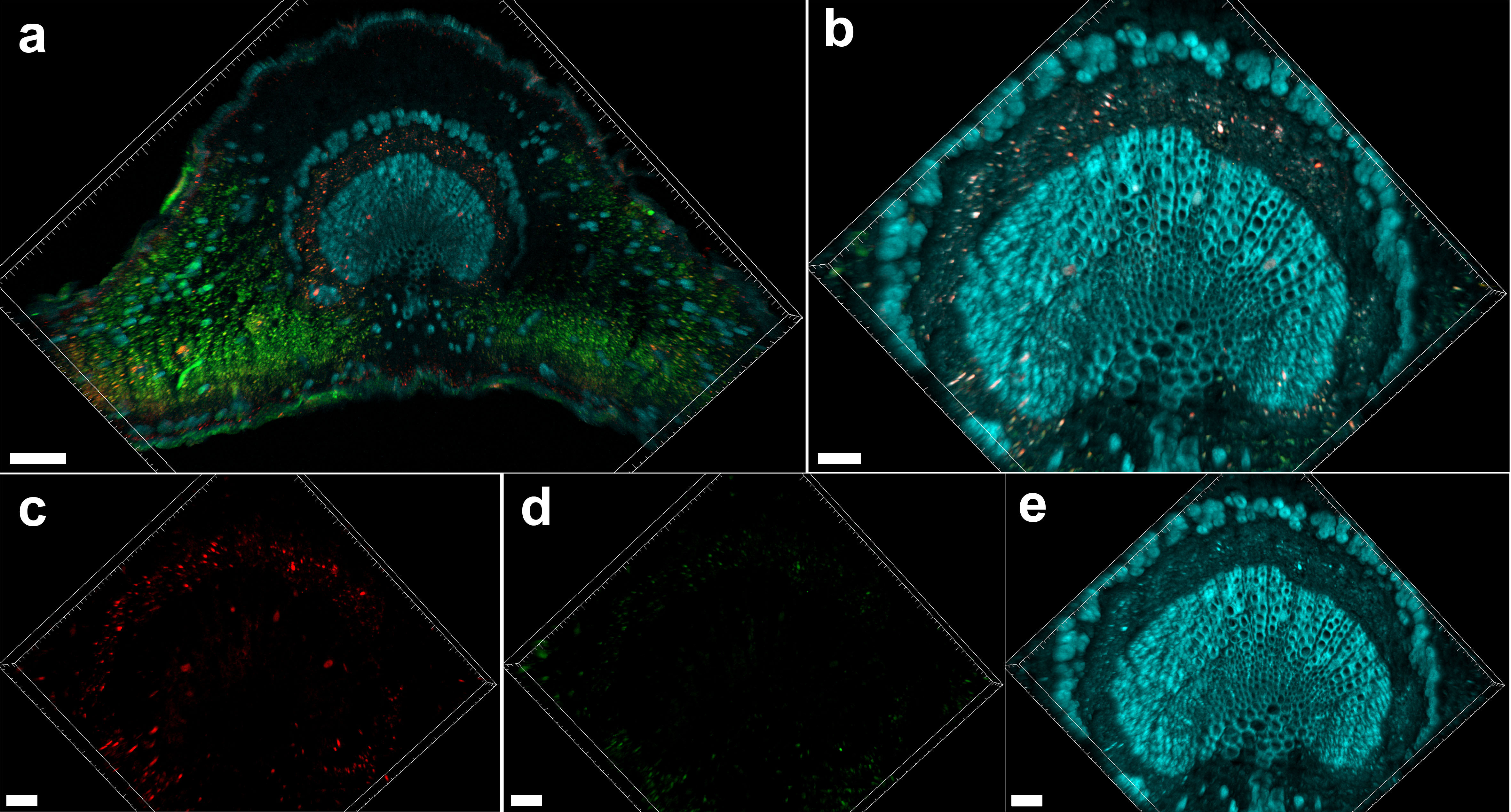

Supplement: FIGURE S2 — Confocal microscopy images, showing the FISH negative control of the basal leaf blade section of a Xylella fastidiosa-infected olive tree. (a) Volume rendering showing only autofluorescence (including the autofluorescence of occluded vessels). (b) Magnification of (a), overlap of (c–e). (c) Signal of the Cy3–NONEUB FISH probe. (d) Signal of the Cy5-NONEUB FISH probe. (e) Plant tissue autofluorescence (scale bars: 150 μm in a, 50 μm in b–e). These are representative images from several observations on five different infected trees. [file Image_2.JPEG]

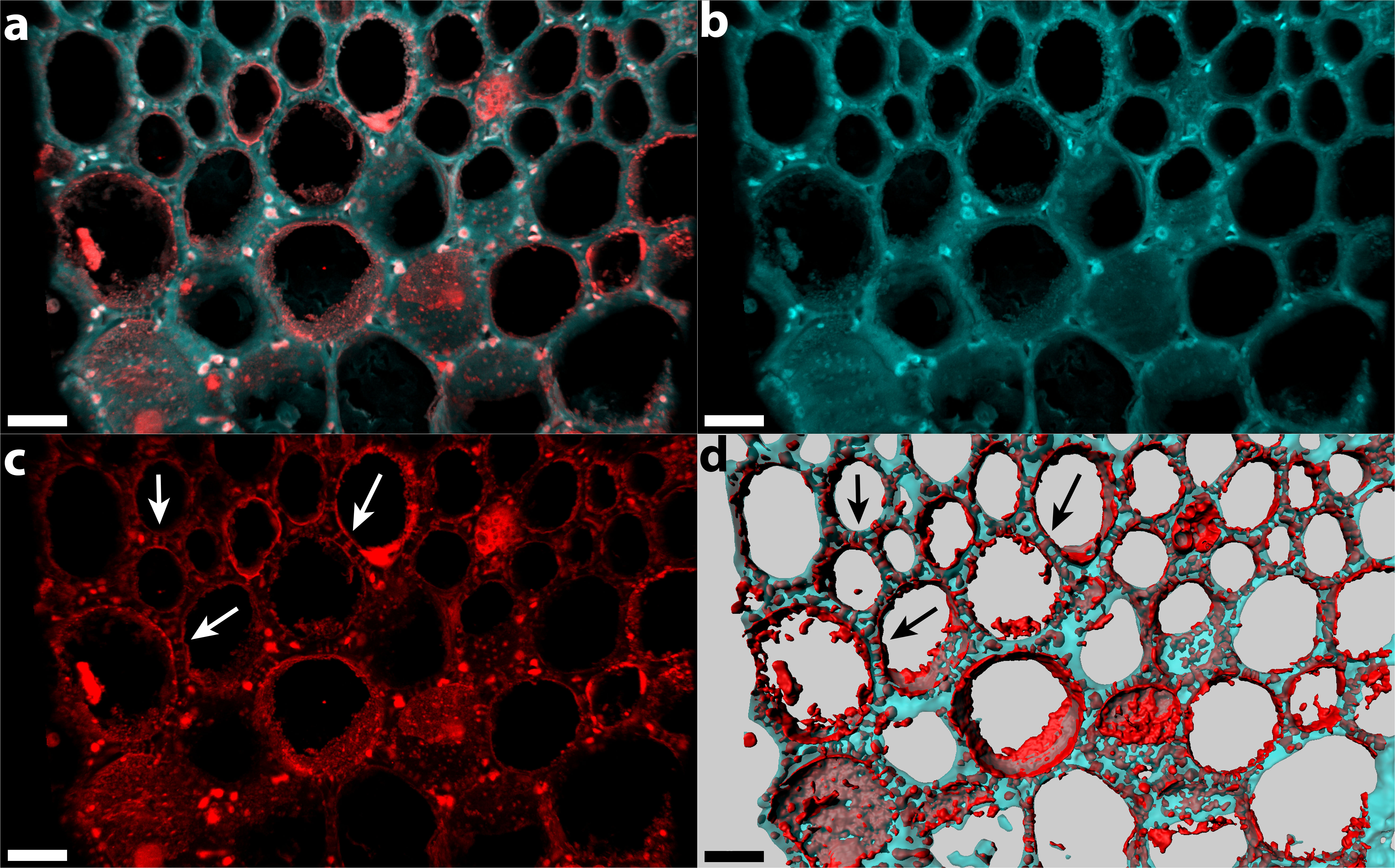

Supplement: FIGURE S3 — Volume rendering of a confocal microscopy stack, showing the autofluorescence of the branch section of a Xylella fastidiosa-infected olive tree. (a) Xylem vessels (cyan) are occluded by an extracellular matrix (red); (a) is the overlap of (b,c). (b) Autofluorescence of the plant vessels. (c) Autofluorescence of the extracellular matrix, which also invaded the lateral connections between adjacent vessels (arrows). (d) Three-dimensional reconstruction of (c). (Scale bars: 20 μm). These are representative images from several observations on five different infected trees. [file Image_3.JPEG]
