# Supplementary material for: Specific Fluorescence in Situ Hybridization (FISH) Test to Highlight Colonization of Xylem Vessels by Xylella fastidiosa in Naturally Infected Olive Trees (Olea europaea L.)
Source: Front Plant Sci. 2018 Apr 6;9:431. doi: 10.3389/fpls.2018.00431 (PMC5897508; doi:10.3389/fpls.2018.00431)
Supplement: TABLE S1 — Alignment between the sequence of the FISH probe KO 210 and all target organisms, as well as all one-mismatch-only, non-target organisms (according to BLAST and ProbeCheck search). [file Table_1.DOCX]

**Tab. S1** Alignment between the sequence of the FISH probe KO 210 and all target organisms, as well as all one–mismatch–only, non–target organisms (according to BLAST and ProbeCheck search).

| **Probe/Organism** | **Accession number** | **Sequence (5’ 🡪 3’)** |
| --- | --- | --- |
| Probe KO 210 |  | GCCACCCATGGTATTACTACC |
| Target organisms |  |  |
| - *Xylella fastidiosa* strain ATCC 35879 | NR_115924 | GCCACCCATGGTATTACTACC |
| - *Xylella fastidiosa* strain GR.8935 | NR_114542 | GCCACCCATGGTATTACTACC |
| - *Xylella fastidiosa* strain PCE-FF | NR_041779 | GCCACCCATGGTATTACTACC |
| Non-target organism used in this work |  |  |
| - *Xanthomonas translucens* strain Mcs_CA2 | MF664205 | GCCACCCATGGTATT**G**CTACC |
| Other Non-target organisms (type strains) |  |  |
| - *Xanthomonas translucens* strain XT 2 | NR_036968 | GCCACCCATGGTATT**G**CTACC |
| - *Xanthomonas albilineans* strain LMG 494 | NR_026316 | GCCACCCATGGTATT**G**CTACC |
| - *Xanthomonas melonis* strain LMG 8670 | NR_026384 | GCCACCCATGGTATT**G**CTACC |
| - *Xanthomonas theicola* strain LMG 8684 | NR_113168 | GCCACCCATGGTATT**G**CTACC |
| - *Dyella humi* strain DHG40 | NR_152698 | GCCACCCATGGTATT**G**CTACC |
| - *Dyella jejuensis* strain JP1 | NR_145542 | GCCACCCATGGTATT**G**CTACC |
